# Evaluation of the Therapeutic Efficacy of Moist Wound Healing After Fractional CO_2_ Laser Surgery

**DOI:** 10.1111/jocd.70079

**Published:** 2025-02-20

**Authors:** Qingmei Jin, Richeng Dong, Jiahui Zhi, Huimin Yin, Meilan Nan, Zhehu Jin, Chenglong Jin

**Affiliations:** ^1^ Department of Dermatology Yanbian University Hospital Yanji China; ^2^ Department of Medical Cosmetology Yanbian University Hospital Yanji China; ^3^ Department of Dermatology Suzhou Mylike Cosmetic Hospital Suzhou China

**Keywords:** fractional CO_2_ laser, moist wound healing, wound healing

## Abstract

**Background:**

With the popularization of laser therapy, an increasing number of patients are undergoing fractional CO_2_ laser therapy. It is particularly important to exercise caution and accelerate wound healing after laser surgery.

**Aims:**

This study aimed to examine the clinical efficacy of moist wound healing after fractional CO_2_ laser therapy.

**Patients/Methods:**

A total of 15 individuals volunteered to undergo fractional CO_2_ laser therapy. The facial skin was irradiated with a fractional CO_2_ laser in the deep mode, with an energy of 15 mJ/cm^2^ and a density of 5%. The left and right sides of the faces were considered the observation and control groups, respectively. After laser therapy, medical cold compress patches were applied on the skin in the control group once a day for approximately 10–15 min, whereas erythromycin ophthalmic ointment was applied on the skin in the observation group 6 times daily for wound care. The wound healing time, duration of erythema, and occurrence of adverse reactions were monitored in both groups.

**Results:**

The observation and control groups received different nursing interventions. The time to regression of erythema and swelling was significantly shorter in the observation group than in the control group. In addition, the scab formed at the wound site was thinner, and shedding was faster in the observation group than in the control group. The Clinical Erythema Assessment score of the observation group was significantly lower than that of the control group (*p* < 0.05). The wound healing time was 5.73 ± 0.70 days in the observation group and 7.73 ± 0.72 days in the control group, with the difference being statistically significant (*p* < 0.05). After 30 min and 12 h of nursing intervention, the Visual Analog Scale score of the observation group was significantly lower than that of the control group (*p* < 0.05). However, after 24 h of treatment, neither group showed significant pain. Both groups showed varying degrees of acne, pustules, and exudation, which subsided within 7 days. Furthermore, the control group had 2 cases of mild pigmentation, which resolved within 3 months. Neither group experienced adverse reactions such as skin infection, depigmentation, or scar formation.

**Conclusion:**

Moist healing therapy can accelerate wound healing and reduce the duration of erythema and edema after fractional CO_2_ laser therapy, demonstrating potential clinical application value.

## 1 Introduction

In aesthetic medicine, it is particularly important to exercise caution and accelerate wound healing after laser therapy. Wound healing is classified as dry and moist [1]. In recent years, the use of moist healing has increased owing to its multiple advantages. However, further investigation is warranted to determine whether its use after CO_2_ laser therapy can improve the healing process. Therefore, this study aimed to evaluate the therapeutic efficacy of moist wound healing after fractional CO_2_ laser therapy.

## 2 Materials and Methods

### 2.1 Ethics Statement

The study was approved by the relevant ethics committee and performed in accordance with the ethical standards stipulated in the 1964 Declaration of Helsinki. All patients have signed the informed consent form for treatment.

### 2.2 Participants

A total of 15 individuals voluntarily underwent fractional CO_2_ laser therapy. The participants comprised eight men and seven women with a mean age of 32 ± 8.30 (range, 23–46) years. The inclusion criteria were as follows: (1) individuals with Fitzpatrick skin types III–IV; (2) individuals with a similar skin condition on both sides of the face; and (3) individuals who had not received laser therapy within the past 6 months. The exclusion criteria were as follows: (1) individuals with facial infections or skin tumors; (2) pregnant or lactating women; (3) individuals with scar constitution or a family history of scar constitution; (4) individuals with a history of allergy to experimental drugs; and (5) individuals with vitiligo, psoriasis, or other conditions that are susceptible to peer reactions during the active period.

### 2.3 Instruments

The Acu Pulse (Lumenis Medical Company, USA) was used to provide fractional CO_2_ laser therapy. The VISIA Skin Image Analyzer (Canfield, USA) and Dermoscopy (Dr. Camscope, Korea) were used to evaluate each patient's skin. The skin was anesthetized with compound lidocaine cream (Tongfang Pharmaceutical Group Co. Ltd., China). Erythromycin ophthalmic ointment (Chenxin Fodu Pharmaceutical Co. Ltd., China) was used to provide moist healing, and a medical cold compress (Guangzhou Chuanger Biotechnology Co. Ltd., China) was used to provide control group care.

### 2.4 Methods

The participants were asked to clean their faces before treatment. The VISIA skin imaging analyzer was used to capture photos and evaluate the facial skin condition of each participant. For local anesthesia, 5% lidocaine cream was applied to the treatment site, and plastic wrap was used to cover the site for 1 h. After local anesthesia was induced, the skin at the treatment site was cleaned and disinfected with iodine. Both sides of the face were treated with a fractional CO_2_ laser in the deep mode, with an energy of 15 mJ/cm^2^ and a density of 5%. The condition of the wound was recorded immediately after the laser treatment. The left and right sides of the face were considered the observation and control groups, respectively. After laser therapy, the wound site in the control group was treated with a medical cold compress once a day for 10–15 min, whereas that in the observation group was treated with the topical application of erythromycin ophthalmic ointment 6 times a day for moist wound healing until complete wound repair was achieved. During the nursing period, the participants were strictly prohibited from exposing the wound to water or forcefully tearing off the scab and were instructed to avoid sun exposure. After treatment, wound healing was observed and recorded daily using the VISIA skin imaging analyzer and dermoscopy until the wound was fully healed, and the participants were followed up for 3 months.

### 2.5 Outcomes

Three doctors who did not participate in the treatment evaluated the treatment outcomes based on images obtained from the VISIA skin imaging analyzer and dermoscopy before and after treatment.

The Clinical Erythema Assessment (CEA) scale was used to evaluate changes in facial erythema before and after treatment in both the observation and control groups. The CEA scores ranged from 0 to 4, indicating no, slight, mild, moderate, and extensive erythema, respectively.

The wound healing time was defined as the time required for the scab at the wound site to completely peel off and for complete epithelialization of the wound base.

### 2.6 Safety Evaluation

After treatment, follow‐ups were continued for 3 months to assess the incidence of postoperative adverse reactions, such as pain, duration of redness and swelling, pustules, bleeding, exudation, pigmentation, hypopigmentation, and scarring.

### 2.7 Statistical Analysis

The Statistical Package for the Social Sciences (SPSS) (version 25.0) software was used for statistical analysis. Normally distributed measurement data were expressed as the mean ± standard deviation. Differences among multiple groups were estimated using one‐way ANOVA, whereas pairwise comparisons were performed using the LSD post hoc test. Measurement data expressed as a rate or percentage were compared using the chi‐squared test, and *p* < 0.05 was considered statistically significant for the difference.

## 3 Results

**FIGURE 1:**
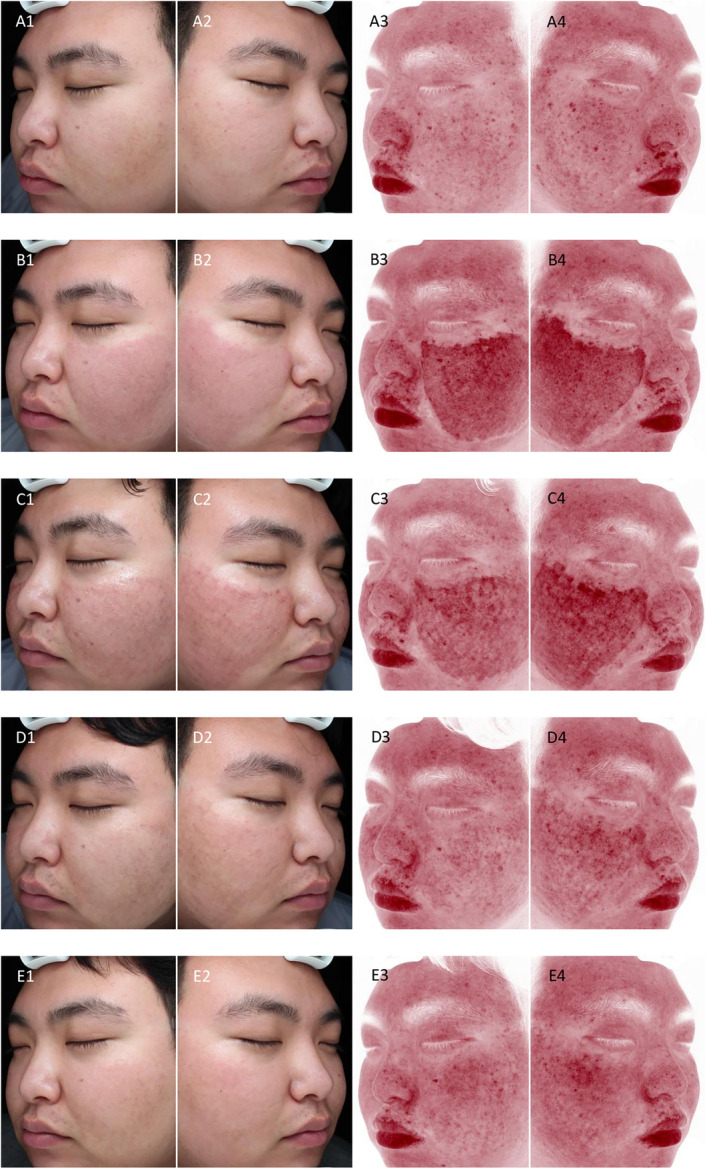
Comparison of wound healing between the observation group and the control group (VISIA skin image analyzer). (A1–A4) at baseline; (B1–B4) immediately postprocedure; (C1–C4) Day 1 postprocedure; (D1–D4) Day 3 postprocedure; and (E1–E4) Day 7 postprocedure.

**FIGURE 2:**
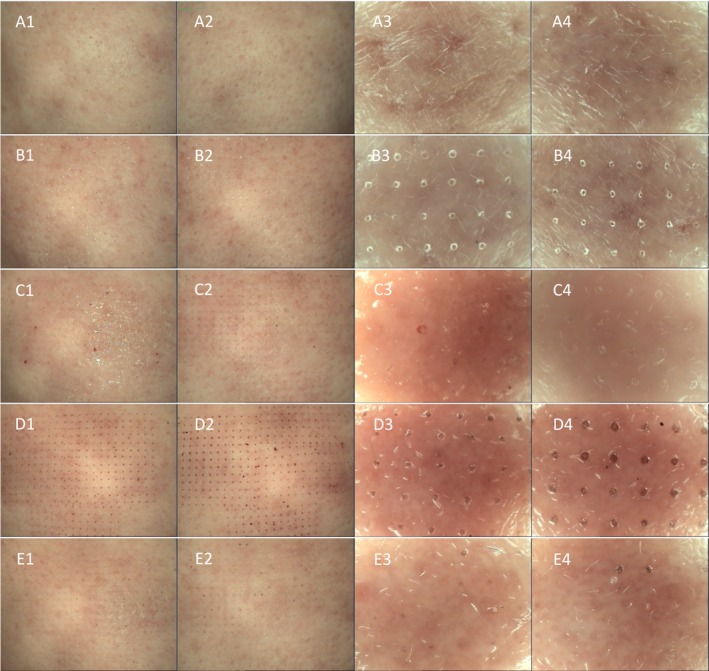
Comparison of scab formation and shedding between the observation group and the control group (dermoscopy). (A1–A4) at baseline; (B1–B4) immediately postprocedure; (C1–C4) Day 1 postprocedure; (D1–D4) Day 3 postprocedure; and (E1–E4) Day 7 postprocedure.

All participants completed the treatment and follow‐up. Images obtained from the VISIA skin image analyzer and dermoscopy showed that both the observation and control groups showed significant erythema immediately after fractional CO_2_ laser therapy, with the degree of erythema being similar in both groups. After the implementation of different nursing interventions, the observation group showed a significantly faster disappearance of erythema and swelling than the control group. In addition, the observation group had a thinner scab and faster shedding than the control group (Figures 1 and 2).

### 3.1 Analysis of Treatment Effectiveness

The CEA scores did not differ significantly between the two groups before or immediately after laser therapy (*p* > 0.05). After nursing intervention for 1, 3, and 7 days, the CEA scores of the observation group were significantly lower than those of the control group (*p* < 0.05) (Table 1). The wound healing time was 5.73 ± 0.70 days in the observation group and 7.73 ± 0.72 days in the control group, with the difference being statistically significant (*p* < 0.05) (Table 2).

**TABLE 1:** Comparison of CEA scores between the observation group and the control group (^−^ *x* ± *s*).

	Baseline	Immediately postprocedure	Day 1 postprocedure	Day 3 postprocedure	Day 7 postprocedure
Observation group	0.13 ± 0.35	4	3.00 ± 0.76	1.93 ± 0.46	0.27 ± 0.46
Control group	0.13 ± 0.35	4	3.80 ± 0.41	2.53 ± 0.52	1.07 ± 0.59
*t*	0		−3.595	−3.367	−4.133
*p*	1		0.001	0.002	0

**TABLE 2:** Comparison of wound healing time and incidence of adverse reactions between the observation group and the control group.

	Wound healing time (days)	Duration of erythema (days)	Duration of edema (days)	Incidence of acne/pustules (%)	Incidence of bleeding/exudation (%)	Incidence of pigmentation (%)
Observation group	5.73 ± 0.70	6.27 ± 0.88	2.73 ± 0.59	53.3 (8/15)	73.3 (11/15)	0 (0/15)
Control group	7.73 ± 0.72	8.33 ± 0.72	3.53 ± 0.83	33.3 (5/15)	26.7 (4/15)	13.3 (2/15)
*t*/*X* ^2^	−6.139	−7.007	−3.027	1.222	6.533	2.143
*p*	0	0	0.005	0.269	0.011	0.143

### 3.2 Analysis of Safety

Immediately after laser therapy, no significant differences in the Visual Analog Scale (VAS) scores were observed between the observation and control groups (*p* > 0.05). After 30 min and 12 h of nursing intervention, the VAS scores of the observation group were significantly lower than those of the control group (*p* < 0.05). However, after 24 h of treatment, neither group had significant pain (Table 3). Erythema and swelling lasted for 6.27 ± 0.88 days and 2.73 ± 0.59 days in the observation group, respectively, and for 8.33 ± 0.72 days and 3.53 ± 0.83 days in the control group, respectively, with the differences being statistically significant (*p* < 0.05). During the nursing intervention, both the observation and control groups developed varying degrees of acne and pustules. The incidence of bleeding and exudation was higher in the observation group than in the control group. All adverse reactions subsided within 7 days in both groups. At the 3‐month follow‐up, the control group had 2 cases of mild pigmentation, which subsided within 3 months. Moreover, neither group experienced adverse reactions such as skin infection, hypopigmentation, or scarring (Table 2).

**TABLE 3:** Comparison of VAS scores between the observation group and the control group (^−^ *x* ± *s*).

	Immediate treatment	After 30 min of treatment	After 12 h of treatment	After 24 h of treatment
Observation group	7.00 ± 1.25	5.73 ± 0.80	2.93 ± 0.59	0.67 ± 0.62
Control group	7.07 ± 1.10	6.47 ± 1.06	3.73 ± 0.59	0.87 ± 0.52
*t*	−0.155	−2.140	−3.691	−0.963
*p*	0.878	0.041	0.001	0.344

## 4 Discussion

Fractional CO_2_ laser therapy, one of the most popular treatments in dermatology, has good therapeutic effects on various skin conditions, such as acne scars, facial fine lines, and enlarged pores [2]. However, the long wound healing time after fractional CO_2_ laser therapy and adverse reactions such as erythema, swelling, and post‐inflammatory pigmentation can lead to scarring if aftercare is not followed strictly. Consequently, patients find it challenging to receive laser therapy [3, 4]. In particular, the cautious administration of postoperative care and the reduction of wound healing time and incidence of adverse reactions in Asians with Fitzpatrick skin types III and IV are some major unaddressed challenges. At present, postoperative care for patients treated with CO_2_ laser therapy primarily involves the use of medical cold compress patches and epidermal growth factor drugs. During the postoperative period, loss of water at the wound site can easily lead to scab formation, and the physical barrier formed by dry tissue, such as scabs, can hinder the process of wound re‐epithelialization and delay healing [5].

Since Dr. George D. Winter demonstrated that wounds healed faster in a moist environment in a pig model, an increasing number of clinicians have used moist wound healing for skin wound care [6]. A moist environment prevents the formation of scabs, allowing wounds to initiate the healing process more quickly [7]. In a moist environment, keratinocytes rapidly migrate to the wound surface to complete re‐epithelialization. However, in a dry environment, they migrate subcutaneously under the scab. Furthermore, a moist environment regulates the partial pressure of oxygen at the wound site and promotes the formation of capillaries, thereby promoting wound healing [1]. A moist environment is also necessary for maintaining cellular function. When the skin is injured, cells communicate with each other by secreting growth factors and other signaling molecules to repair tissues in an orderly manner. These molecules require a liquid medium for intercellular crosstalk to promote healing [5, 8, 9]. A moist environment retains the tissue protease enzymes contained in the exudate, which can promote the dissolution and absorption of necrotic tissue and alleviate pain [10]. In addition, it alleviates inflammation at the wound site and reduces the risk of scar formation [10, 11].

A moist environment does not increase the risk of infection at the wound site, as it effectively isolates the wound from external contact. Some studies have shown that the bacterial content of wounds treated with closed moist dressings is lower than that of wounds treated with dry dressings. On the contrary, dry crusts contain a high load of biofilm bacteria [12], and the defense mechanisms of body fluids and tissues are more active in moist environments.

In our study, we used erythromycin ophthalmic ointment to accelerate wound healing after fractional CO_2_ laser therapy. Erythromycin is a macrolide antibacterial drug, and its ophthalmic ointment mainly consists of erythromycin, liquid paraffin, lanolin, and yellow Vaseline. Yellow Vaseline is a widely used moisturizer that can reduce the transdermal water loss rate of damaged skin [13], and liquid paraffin has been shown to accelerate the healing of burns [14]. Erythromycin ophthalmic ointment not only provides a moist environment for wounds but also has a low risk of causing adverse reactions. Using medical cold compresses after fractional CO₂ laser treatment on the face is a commonly used nursing method in current clinical practice [15]. Therefore, based on our previous clinical experience, we selected medical cold compresses as the intervention for the control group.

In this study, a total of 15 participants received fractional CO_2_ laser therapy and were followed up for 3 months. The results showed that the observation group (which received moist wound care) had fewer scabs and faster shedding than the control group (which received dry wound care). The degree of erythema, duration of swelling, and severity of pain were significantly lower in the observation group than in the control group. Both groups showed varying degrees of acne and pustules after treatment, which are common adverse reactions after fractional CO_2_ laser therapy. However, the incidence of acne and pustules was higher in the observation group than in the control group. We speculate that the oily texture of erythromycin ophthalmic ointment promoted the formation of acne and pustules in the observation group. In an effort to minimize the occurrence rate of acne and pustules resulting from moist care, specifically for patients with oily skin, it is advisable that we reduce the quantity of erythromycin ophthalmic ointment being applied and duly cut down the frequency of its application. In addition, if acne and pustules occur, simple needle extraction treatment can be carried out to accelerate the recovery. In the early stages of moist wound healing, the blood and tissue fluid oozing from the wound prevented the formation of scabs, resulting in higher exudation than that in the control group, which is a normal manifestation of moist healing. During the 3‐month follow‐up, 2 cases of pigmentation were observed in the control group; however, no adverse reactions, such as pigmentation, hypopigmentation, or scars, were observed in the observation group, but the difference was not statistically significant. This non‐significant difference may be attributed to the small sample size and the relatively conservative use of laser energy. For clinical doctors, we recommend widely applying the moist wound care method to the wound care in the treatment of diseases such as acne atrophic scars, hypertrophic scars, enlarged pores, and photoaging using fractional CO_2_ laser therapy, so as to shorten the wound healing time and reduce the occurrence of adverse reactions. In future studies, the sample size should be increased and the parameters of laser therapy should be improved to accurately evaluate the clinical efficacy of moist wound healing and the incidence of adverse reactions after laser therapy. In addition, since all the subjects in this study are Asians, the results of this study are applicable to the Asian population with Fitzpatrick skin types III–IV. For populations with other skin types, further observational research is required.

## 5 Conclusion

The results of this study suggest that moist wound healing accelerates the healing process and reduces the duration of erythema and swelling after fractional CO_2_ laser therapy. Therefore, the clinical application of moist wound healing should be expanded.

## Author Contributions

Q.J. and R.D. designed the study, analyzed the data, and wrote the manuscript. J.Z., H.Y., and M.N. contributed to collection of clinical of data. Q.J., R.D., and C.J. reviewed and revised the manuscript. Z.J. and C.J. conceived of and supervised the study. All authors have read and approved the final manuscript.

## Ethics Statement

The study was performed in accordance with the ethical standards stipulated in the 1964 Declaration of Helsinki and was approved by the Medical Ethics and Human Research Committee of Suzhou Mylike Cosmetic Hospital of China. All patients provided written informed consent. Ethical No. 2020002.

## Consent

A total of 15 individuals who volunteered to undergo fractional CO_2_ laser therapy were recruited from Suzhou Mylike Cosmetic Hospital and Yanbian University Hospital.

## Conflicts of Interest

The authors declare no conflicts of interest.

## Data Availability

The data that support the findings of this study are available from the corresponding author upon reasonable request.
